# Reactivation of Bovine Tuberculosis in Patient Treated with Infliximab, Switzerland

**DOI:** 10.3201/eid1507.090024

**Published:** 2009-07

**Authors:** Marina Nager, Philip E. Tarr, Horst G. Haack, Ferdinand Martius, Claudio Stoebe, Reno Frei, Juerg Danuser, Andreas W. Jehle

**Affiliations:** University of Basel, Bruderholz, Switzerland (M. Nager, P.E. Tarr, H.G. Haack, F. Martius, C. Stoebe, A.W. Jehle); University Hospital Basel, Basel, Switzerland (R. Frei); Swiss Federal Veterinary Office, Berne, Switzerland (J. Danuser)

**Keywords:** Mycobacterium bovis, bovine, tuberculosis and other mycobacteria, infliximab, reactivation, dairy products, tumor necrosis factor, Crohn’s disease, letter

**To the Editor:** Increased risk for reactivation of tuberculosis (TB) after treatment with tumor necrosis factor (TNF) antagonists, particularly infliximab, is well documented (*1*). We describe a case of peritoneal TB, probably resulting from reactivation of *Mycobacterium bovis* infection after infliximab treatment. In retrospect, reactivation might have been preventable had physicians been aware of the patient’s history of regularly drinking fresh cow’s milk from a local farm in Switzerland during 1944–45, when bovine TB was prevalent.

The patient was a 69-year-old Swiss woman who was examined for weakness, abdominal pain, increasing abdominal girth, and weight loss in April 2008. Her history included a diagnosis of Crohn disease in 1978 and treatment with glucocorticoids, cyclosporine, and mercaptopurine. In November 2007, during a recurrence of Crohn disease and while receiving treatment with azathioprine, the patient had a negative interferon gamma release assay (IGRA) result (QuantiFERON-TB in Tube test; Cellestis International, Carnegie, Victoria, Australia) and an unremarkable chest radiograph. On November 30, 2007, she was given prednisone (40 mg/d for 2 weeks, then tapered) and prescribed 3 doses of infliximab because of severe inflammation seen during colonoscopy (5 mg/kg on November 30, 2007, January 4, 2008, and February 15, 2008). She was hospitalized on April 25, 2008, at which time she had ascites; the fluid contained 1.4 × 10^9^/L leukocytes (79.5% lymphocytes) but was negative for acid-fast bacilli on direct examination and PCR testing for *Mycobacterium tuberculosis* complex. Laparoscopy on May 2, 2008, showed extensive peritoneal inflammation. Peritoneal biopsy samples contained acid-fast bacilli and caseating granulomas; PCR for *M. tuberculosis* complex was positive. At this time, results of a repeat QuantiFERON-TB in Tube test and a tuberculin skin test (TST) were negative, but a T-SPOT.TB (Oxford Immunotec, Abingdon, UK) test was positive (6-kDa early secretory antigenic target [ESAT-6], >20 spots; 10-kDa culture filtrate protein [CFP-10], 11 spots). *M. bovis* ssp. *bovis* was grown in cultures of peritoneal biopsy samples. For culture, the MGIT 960 automated culture system (Becton Dickinson, Sparks, MD, USA) was used. The isolate was identified by use of a multiplex PCR-based, solid-phase, reverse-hybridization assay (GenoType MTBC, Hain Lifescience GmbH, Nehren, Germany), excluding *M. bovis* BCG (*2*). The patient was discharged May 30, 2008. In January 2009, she was much improved after treatment with isoniazid/rifampin/ethambutol for 3 months and moxifloxacin/rifampin for 5 months.

This case of presumed reactivation of peritoneal TB caused by *M. bovis* in a patient treated with infliximab highlights the need to be aware of local epidemiology with regard to transmissible infectious diseases. It particularly emphasizes the value of careful history taking regarding consumption of unpasteurized dairy products in assessing risk for latent TB infection (LTBI) before starting anti-TNF treatment. Some infectious diseases experts (*3*) recommend considering LTBI treatment before prescribing anti-TNF therapy even when TST is negative or only minimally positive (<5 mm induration) for patients with epidemiologic or clinical hints of LTBI. This case indicates that this recommendation should be extended to assessment of the probability of latent *M. bovis* infection. This decision may not be easy, and referral to a TB specialist should be considered. Excluding LTBI on the basis of a negative TST result or IGRA is problematic because of the limited sensitivity of these tests, especially for immunosuppressed patients (*4*). Of note, the antigens used in IGRAs are present in *M. bovis*. Discordant TST and IGRA results are frequent, and these differences are not easily explained (*4*). However, IGRAs are not confounded by prior vaccination with *M. bovis* BCG, and the T-SPOT.TB test may have a higher sensitivity than QuantiFERON-TB and QuantiFERON-TB Gold tests (Cellestis International) (*5**,**6*). Our patient had a positive T-SPOT.TB result and negative QuantiFERON-TB and TST results at the time of her diagnosis of peritoneal TB.

Recently, another case of peritoneal TB due to *M. bovis* in a patient treated with infliximab was reported in Denmark. This patient’s exposure presumably consisted of consumption of unpasteurized milk at a dairy (*7*). Tuberculosis cases caused by *M. bovis* are probably underreported because many laboratories determine only whether a pathogen belongs to *M. tuberculosis* complex. In the United States, genotyping of culture-confirmed *M. tuberculosis* complex has been routinely done only since 2004 (*8*).

Our patient probably acquired *M. bovis* infection by frequent consumption of unpasteurized milk in a rural area of Switzerland during the 1940s. Infection by the respiratory route is conceivable but unlikely. As the patient traveled rarely, and only in industrialized countries, more recent transmission of *M. bovis* seems unlikely. According to the requirements of section 3.2.3.10 of the World Organisation for Animal Health International Animal Health Code, Switzerland has officially been free from bovine TB since 1959. The last sporadic case occurred in 2003, although eradication has not been achieved in all neighboring European countries (*9*). Of note, in the United States and Canada, where *M. bovis* has been virtually eliminated in cattle (*10*), *M. bovis* infection continues to be diagnosed today, particularly in children of Hispanic origin and those born outside the United States. The principal infection route may be consumption of unpasteurized dairy products from Mexico (*8*). Awareness of the epidemiology of bovine TB and careful history taking regarding recent or distant (in countries where *M. bovis* infection in cattle has been eradicated) consumption of unpasteurized dairy products may prompt preventive chemotherapy before the start of anti-TNF treatment. Thus, a potentially increasing number of human TB cases due to *M. bovis* may be prevented*.*
